# Pilot Implementation of HIV Self-Testing Delivery in Private Pharmacies Combined With a Respondent-Driven Sampling Method to Improve HIV Testing for Men Who Have Sex With Men and Transgender Women in Phnom Penh (ANRS 0100s): Protocol for a Prospective Mixed Method Feasibility Study

**DOI:** 10.2196/65351

**Published:** 2025-06-27

**Authors:** Chhavarath Dary, Olivier Ségéral, Joseph Larmarange, Emilie Mosnier, Mohamed Ben Mechlia, Vichea Ouk, Bruno Spire, Vonthanak Saphonn

**Affiliations:** 1 Public Health Unit University of Health Sciences Phnom Penh Cambodia; 2 HIV Unit Division of Infectious Diseases, University Hospital Geneva University of Geneva Geneva Switzerland; 3 Centre Population et Développement Université Paris Cité Institut de Recherche pour le Développement, Université Sorbonne Paris Nord, Inserm Paris France; 4 Emerging Infectious Diseases French National Agency for Research on AIDS and Viral Hepatitis University of Health Science Phnom Penh Cambodia; 5 Aix Marseille Univ IRD, ISSPAM, Inserm Marseille France; 6 Department of Research in Public Health and the Human and the Social Science French National Agency for Research on HIV/AIDS , Viral Hepatitis, Tuberculosis, Sexually Transmitted Infections, and Emerging Infectious Diseases Paris France; 7 National Center for HIV/AIDS, Dermatology and STD Phnom Penh Cambodia

**Keywords:** feasibility study, HIV self-test, MSM, TGW, respondent-driven sampling, private pharmacies, Cambodia, transgender women

## Abstract

**Background:**

Regular testing is recognized as a key strategy for HIV control. The 2023 Integrated Biological and Behavioral Survey (IBBS) in Cambodia revealed that nearly one-third of men who have sex with men (MSM) and one-fourth of transgender women (TGW) were never tested for HIV or not for more than 12 months. The majority of MSM and TGW were tested at community-based organizations (CBOs) facilities and by CBO outreach workers, while private facilities are poorly used for HIV testing (6% for MSM and 9% for TGW). Private pharmacies could be able to deliver HIV self-testing kits giving the advantage of confidentiality, anonymity, and time savings, in particular for those reluctant to visit CBOs. The recruitment of participants using a respondent-driven sampling method could provide the opportunity to reach MSM and TGW outside the network of CBOs.

**Objective:**

This pilot study aims to evaluate the feasibility of free HIV self-testing delivery by a private pharmacy combined with a respondent-driven sampling method to improve HIV testing among MSM and TGW in Phnom Penh, Cambodia.

**Methods:**

Both qualitative and quantitative approaches are used in this prospective feasibility study. The protocol was approved by the National Ethics Committee for Health Research in Cambodia (N0 351 NECHR). MSM and TGW aged more than 18 years old will be recruited via a respondent-driven sampling method with seeds recruited at hot spots and on social networks. The seeds will then distribute electronic and paper coupons to their networks physically and via social media, messaging, and calling applications. Each recruited peer will bring the coupon to receive direct and free access to one HIV self-testing kit at partner pharmacies as well as 10 additional coupons to recruit members of their networks. As for quantitative analysis, data from the study website will be imported, appended into a single matrix using Stata version 18SE (StataCorp), and analyzed using descriptive statistics, with a statistical significance level of .05. After 6 months, a qualitative assessment will be conducted among users, providers, and policymakers or key stakeholders to evaluate the acceptability and appropriateness of the strategy and to identify the barriers, facilitators, and recommendations. All transcripts will be analyzed according to the 6-phase reflexive thematic approach by Braun and Clarke. The results will be reported by participant-based and provider-based issues. QSR NVivo V.14 for Windows will be used to manage the data.

**Results:**

The study was funded by Agence nationale de recherches sur le sida, les hépatites, et les maladies infectieuses émergentes (ANRS) in September 2023. Approval of the study protocol was successfully obtained from the NECHR in Cambodia and the Commission Nationale de l'Informatique et des Libertés (CNIL) in France (Autorisation Tacite) in February 2025. Data collection will be conducted between September 2024 and December 2025. The initial results are expected to be published in February 2026.

**Conclusions:**

This public-private partnership intervention could allow the hidden population of MSM and TGW in Phnom Penh to be reached and tested.

**Trial Registration:**

ClinicalTrials.gov NCT05745168; https://clinicaltrials.gov/study/NCT05745168

**International Registered Report Identifier (IRRID):**

PRR1-10.2196/65351

## Introduction

### Background

Regular testing is acknowledged as a critical approach in HIV management. Knowing one’s HIV-positive status leads to a decrease in high-risk sexual behaviors, prompt connection to health care services, early commencement of antiretroviral therapy (ART), and a significant decrease in the likelihood of transmitting HIV to sexual partners. Over the last 10 years, HIV testing services have considerably expanded [1].

The sharp rise in the number of new HIV infections among men who have sex with men (MSM) has continued in Asia, and sexual activity between men remains stigmatized and frequently hidden. HIV prevalence among MSM was 5% or higher in 10 of the 24 countries that reported these statistics to the Joint United Nations Programme on HIV/AIDS in 2019 [2]. Approximately 30% of all new infections in Asia are in MSM, and HIV incidence is high among young (15-24 years old) MSM [2]. In Southeast Asia, HIV prevalence and incidence continue to be elevated, especially among younger MSM [3]. For instance, a recent publication reported an HIV incidence of 7.4 per 100 person-years in Bangkok in this population [4]. Consensus was that reduction in HIV prevalence and incidence has not been observed among transgender women (TGW) in the region [3,5]. The implementation of preventive ART and HIV preexposure prophylaxis (PrEP) should have led to a reduction. Nonetheless, HIV prevalence and incidence remain high, with no indications of a downturn among young MSM and TGW in Southeast Asia [6]. Given the current rate of new HIV infections among MSM and TGW, the region is unlikely to achieve the target of eradicating AIDS by 2030.

A number of studies have been reported regarding HIV surveillance, HIV and sexually transmitted infection prevalences, population size estimations, and behavior patterns for either TGW or transgender men in the Asia Pacific region. The data show disturbing HIV infection rates throughout the region: Delhi: 49%; Mumbai: 42%; Phnom Penh: 37%; Jakarta: 34%; Surabaya: 25%; Bandung: 14%; Chiang Mai: 18%; Phuket: 12%; Bangkok: 11%; and Lahore: 0.5% [7].

Late diagnosis is a serious barrier to tackling HIV. Approximately 69% of people living with HIV in the Asia and Pacific region were aware of their status in 2018, up from 58% in 2015. However, this means approximately 1.9 million people did not know they were HIV positive. Progress on testing varies greatly between countries. In Thailand, 94% of people living with HIV were aware of their status in 2018, as were 86% of people living with HIV in Malaysia and 82% of people living with HIV in Cambodia. Stigma, discrimination, and punitive legal environments prevent many people from key populations from accessing testing services [8]. Indeed, in Malaysia and Sri Lanka, the rates of HIV testing and awareness among MSM are 43.3% and 40.3%, respectively, while the rates of HIV testing and awareness for TGW people are 43% and 36.9%, respectively [9].

Although Cambodia admirably achieved the 90-90-90 targets by 2020, there is still cause for concern regarding the HIV epidemic among key populations and unidentified priority populations. Compared with data from the 2019 Integrated Biological and Behavioral Survey (IBBS) survey, the 2023 IBBS key population data show an increase in HIV prevalence, from 4% to 5.5% among MSM and from 9.6% to 13.5% among TGW [10,11]. Despite these high prevalence rates, the uptake of HIV testing among MSM (<50%) and TGW (39%) remains low. Moreover, consistent condom use for anal sex with noncommercial sexual partners is suboptimal, at 69% for MSM and 38% for TGW [12]. Although PrEP has been found to protect against HIV infection in high-risk populations, the country struggled to reach the target PrEP enrollment rate (10,000 users by 2023) among MSM and TGW [13]. The 2023 IBBS report highlighted that nearly one-half of MSM and one-fourth of TGW did not report any HIV testing or not for more than 12 months. This suggests that undiagnosed HIV infections may persist in hard-to-reach MSM and TGW and new modalities of HIV testing approaches are hence needed to improve the uptake of HIV testing among these key populations. In addition, of those who reported HIV testing, the majority of MSM and TGW were tested at community-based organization (CBO) facilities (70.1% for MSM and 74.1% for TGW) and by CBO outreach workers (9% for MSM and 6% for TGW), while private facilities are poorly used for HIV [14].

From the perspective of the general population as well as some users from “hidden” or “marginalized” groups such as MSM and TGW, HIV self-test results should be interpreted in a private setting to ensure complete anonymity. HIV self-testing is a process in which a person performs an HIV rapid diagnostic test and interprets the result in private. Lay users can perform HIV self-testing reliably and accurately, achieving a performance comparable with that of trained medical professionals [15]. HIV self-testing has the potential to increase the number of people living with HIV who have access to testing, know their status, are diagnosed, and initiate treatment. HIV self-testing shares many characteristics with current HIV testing and counseling approaches, including products, accuracy issues, linkage to care, potential benefits and risks, and regulatory policies and frameworks [16]. Of some key populations, HIV self-testing increased testing frequency among high-risk MSM and could increase HIV diagnoses in the trans community [17,18]. In 2016, the World Health Organization (WHO) recommended HIV self-testing as a safe, accurate, and effective way to reach people who may not test otherwise, including people from key populations, men, and young people [15]. Representatives from 13 countries across Asia and the Pacific gathered to develop road maps to implement HIV self-testing in the region [19].

In 2017, Cambodia developed national consolidated guidelines on HIV testing services in which many approaches including HIV self-testing are recommended to increase HIV testing uptake, especially among hard-to-reach and key populations [20]. Recent evaluation of the Boosted-Integrated Active Case Management in Cambodia revealed that 66.5% who received assisted HIV self-testing wanted to confirm their HIV status [21]. Even though MSM and TGW had not heard about HIV self-testing, all of them expressed a willingness to try it [22].

Although HIV self-test kits are distributed primarily from public health facilities, HIV self-tests are also available through private ways and channels. Some studies, including the one conducted in Cambodia by Pal et al [22], suggest that MSM and TGW wish or prefer HIV self-tests to be available over the counter at pharmacies and other locations or through the internet [23]. HIV self-testing is already formally and informally available, and it will likely become increasingly available. Indeed, HIV self-test kits are authorized to be dispensed from pharmacies in some countries such as the United States and France [24,25]. In addition, HIV self-tests were reported informally on sale through the internet and in pharmacies in many countries—with specific reports on this from Australia, China, Namibia, Peru, South Africa, Philippines, and Malaysia [26].

Recent studies have also shown that the take-up rate for HIV testing was high, with most participants preferring oral fluid testing to finger prick testing. Many people (72%) who had never accessed HIV testing services took part in the test, with high reactivity rates [27].

Respondent-driven sampling, a form of peer referral–based sampling, has become a popular strategy to recruit “hidden” or “marginalized” populations such as MSM and TGW [28]. In Cambodia, this type of method is widely used and highly effective at reaching these populations [29,30]. To mitigate some of the challenges of implementing the methodology, such as notably slow recruitment rates, innovation around conventional respondent-driven sampling can be helpful [31]. Indeed, since there has been an increase in the use of the internet and online communities by gay and bisexual men in the last few decades to facilitate new connections for the purpose of information seeking, socializing, and seeking sex, several studies have reported innovative internet- and application-based respondent-driven sampling strategies in different aspects of HIV/AIDS research such as improving uptake of HIV self-testing among MSM; providing technical support, counseling, and referrals for further HIV testing services; and HIV prevention, care and treatment, and other services [32-37].

The main hypothesis of this study is that private pharmacies could deliver HIV self-testing kits, given the advantage of confidentiality, anonymity, and time savings, and this could be an effective intervention to improve HIV testing coverage for MSM and TGW. The second hypothesis is that a respondent-driven sampling method with a diversity of seeds recruited at hot spots and on social networks could help to identify a new network of a hidden key population.

Given the persistence of high HIV prevalence and incidence rates among MSM and TGW in southeast Asia, despite advancements in preventive treatments and PrEP, there is an urgent need to re-evaluate and innovate our strategies toward HIV testing and awareness. The complexities inherent in the sociocultural fabric of this region, alongside logistical challenges, underscore the necessity for localized, community-driven interventions that are sensitive to the unique needs and challenges faced by these key populations.

### Primary Objective

The primary objective is to evaluate the feasibility of HIV self-test delivery by a private pharmacy network among MSM and TGW recruited through classic and digital respondent-driven sampling methods to improve HIV testing in Phnom Penh, Cambodia.

### Secondary Objectives

The secondary objectives are to evaluate the acceptability and appropriateness of the strategy, identify barriers and facilitators, estimate the linkage to confirmatory testing for those with a reactive test and linkage to HIV care and ART for those with a positive confirmatory HIV test, estimate linkage to PrEP services for negative participants, identify the characteristics of participants and compare with those reported in IBBS 2023 for MSM and TGW in Cambodia, and evaluate the adherence of participants to 6-monthly repeated HIV testing.

## Methods

### Ethical Considerations

The National Ethics Committee for Health Research (NECHR) in Cambodia provided ethical approval for this ANRS-0100s study (number 351 NECHR).

This study emphasizes voluntary patient participation, with comprehensive information provided by the research team about the study’s objectives, procedures, timeline, potential risks and benefits, and any associated discomfort. Information is conveyed both orally and in written form in the Khmer language. Consent covers several key aspects, including overall participation, compensation, potential phone recontact, and participation in focus group discussions, necessitating prior informed consent before any study-related assessments. Participants also have the option to retain a copy of the consent form after signing. Additionally, the study integrates community-based workers to ensure nonstigmatizing, high-quality, and participant-tailored reception. To guarantee privacy and confidentiality, each participant is assigned a pseudonym or unique code (eg, NBZ0081@) that does not constitute personally identifiable information. Participants will receive incentives of US $2 for successfully returning their results and US $1 per recruited peer. For those participating in focus group discussions for the qualitative assessment, the study will cover transportation expenses for their time and contribution.

### Study Design

A pilot study using a mixed qualitative and quantitative approach will be carried out to assess the uptake of HIV self-test delivery by a private pharmacy network among MSM and TGW recruited through a respondent-driven sampling method to improve HIV testing in Phnom Penh, Cambodia.

The participant recruitment method will use respondent-driven sampling by recruiting diverse seeds both at hot spots and on social networks.

After 6 months, a qualitative assessment of focus group discussions will be conducted among MSM, TGW, and pharmacists to evaluate the feasibility, acceptability, and appropriateness of the intervention and to identify barriers and facilitators.

### Study Site

**Figure 1:**
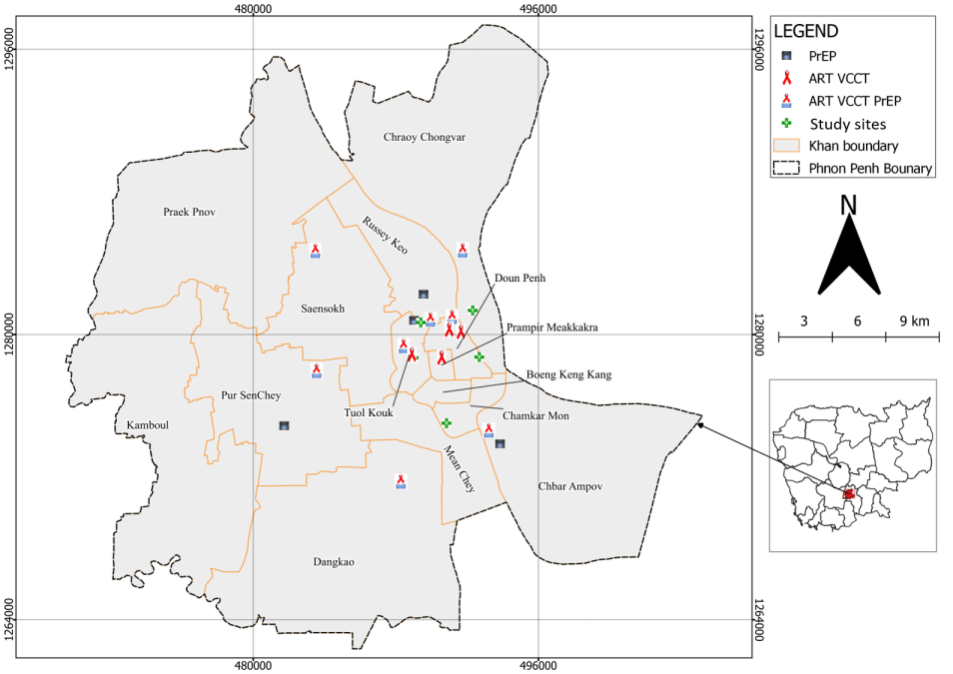
The 3 to 5 private pharmacies with their respective antiretroviral therapy (ART), voluntary HIV counseling and testing (VCCT), and preexposure prophylaxis (PrEP) sites in Phnom Penh that support the follow-up among study participants who report HIV self-test results.

The study sites are located in Phnom Penh, specifically at 3 to 5 private pharmacies selected based on factors such as working hours, availability of dedicated space, and geographical balance. Furthermore, ART; voluntary, confidential counseling and testing; and PrEP sites in Phnom Penh will serve as the study participants’ follow-up contacts after they have self-tested for HIV (Figure 1).

### Study Population and Sample Size

MSM and TGW who are residing in Phnom Penh at the time of enrollment and meet the eligible criteria constitute the study population. The inclusion criteria are being at least18 years old (legal age in Cambodia); for MSM, to have at least one oral or anal intercourse with another man in the past 12 months; and for TGW, to be biologically male at birth, self-identify as a woman or third gender, and have at least one oral, anal, or vaginal intercourse with another man in the past 12 months.

We will use a pragmatic approach with the objective of recruiting the maximum numbers of MSM and TGW. According to IBBS 2019, the estimated number of MSM in Phnom Penh is 6300, and the estimated number of TGW is 1400. With 1500 participants (1000 MSM and 500 TGW), we will be able to estimate an HIV self-test uptake of 70% to within a 95% CI of +/- 2% to 2.5% (width of the confidence interval [in %]=1.96 x √(p x (1-p) / n)).

### Procedures

#### Respondent-Driven Sampling Implementation

Implementation of respondent-driven sampling resembles snowball sampling with several critical caveats. Initial participants are purposefully recruited to be “seeds” as long as they fit the study’s eligibility criteria. After completing the study procedures, seeds are offered a limited number of vouchers to recruit their peers to participate. In this study, two categories of vouchers will be used: paper and electronic vouchers. The latter was adopted for the main advantage that they could be passed electronically to the social networks of respondents regardless of geographical coverage [38]. When vouchers are redeemed, eligible participants also complete the same study procedures and are asked to recruit their peers, and this continues until recruitment goals are met. Using specially formulated statistical programs, sampling weights are developed and applied to estimate population parameters. For the purpose of respondent-driven sampling, effective seeds generate large recruitment chains and samples, which has been associated with motivation and a commitment to the research goals [27].

**Figure 2:**
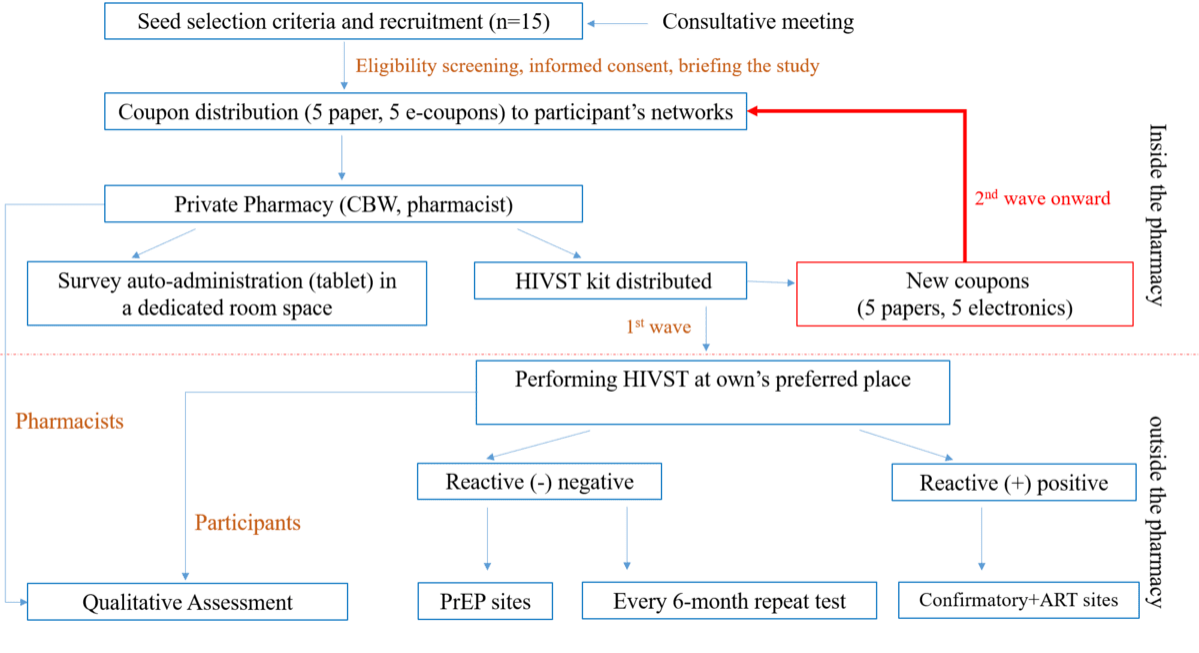
Study flow depicting participant recruitment by respondent-driven sampling, follow-up for HIV self-test results, willingness to repeat the test every 6 months, and qualitative study. ART: antiretroviral therapy; CBW: community-based workers; HIVST: HIV self-test; MSM: men who have sex with men; PrEP: preexposure prophylaxis; TGW: transgender women.

The study sites include 3 to 5 private pharmacy outlets in Phnom Penh with a proper dedicated room or space that secures privacy and secrecy. Pharmacists will be trained by the research teams about the study objectives, study procedures, communication with MSM and TGW, and counseling. We will recruit 15 diverse seeds both at hot spots and on social networks based on optimized criteria obtained from meeting with stakeholders and CBOs. Once consented to the study, the pharmacy provides the seed with a free HIV self-testing kit and two types of coupons (5 electronic coupons [e-coupons] and 5 paper coupons) to distribute to their networks either physically or via social media messaging, and calling applications such as Twitter, WhatsApp, and Telegram (e-coupons). Each recruited participant, so-called peers, will bring the coupon to be scanned at partner pharmacies to receive direct and free access to one HIV self-test kit. There, the pharmacist will notify a trained community worker or the research team of the participant visit; the community worker or research team member will conduct the study visit, including the informed consent process, explaining how to complete the questionnaire on the tablet (Multimedia Appendix 1), and providing the link to an external website to submit the HIV test results and obtain necessary support like tutorials and follow-up after receiving their HIV results. These recruited individuals will be considered wave 1 of recruitment and will each receive 10 additional coupons to recruit members of their networks. The next round of individuals recruited and enrolled will be considered wave 2 and so on. To return the HIV self-test results, participants, who will be provided a unique username and password, will upload a picture of the results labeled with their corresponding username to the external website that they received from the community worker or research team during their pharmacy visit. A token or incentive is granted to those who return the HIV self-test results. Each nonreactive participant will be provided another new coupon (paper or electronic format upon their preference) with a reminder to perform 6-monthly HIV tests for a period of 18 months. The study flow is represented in Figure 2.

#### Qualitative Assessment

After 6 months, a qualitative assessment via focus group discussion (6-8 participants) among users (MSM and TGW) and providers (pharmacists) will be conducted to evaluate the feasibility, acceptability, and appropriateness of the intervention and to identify barriers and facilitators of this pilot intervention.

Participation in the focus group discussion and key informant interviews will be anonymous and voluntary. The interviews will be conducted in Khmer by trained researchers. Semistructured interview guides for focus group discussions among MSM and TGW emphasize their perception, the acceptability, and the appropriateness of their HIV self-test experience including facilitators and barriers and intention to promote and retake the HIV self-test (Multimedia Appendix 2). Focus groups will be conducted between participants and the trained research team in a dedicated room. Each focus group will be composed of 9 to 10 members for a 60-minute to 90-minute discussion that will be recorded anonymously to facilitate retranscription and translation. Records will be deleted at the end of the study.

As for the focus group with pharmacists, the guide covers the following topics: perception, acceptability and appropriateness, improvement, and willingness to sustain the strategy of HIV self-test distribution in private pharmacies (Multimedia Appendix 3). Regarding the in-depth interview with policymakers or key stakeholders involved in HIV programs, the interview guide aims to explore perceptions of the strategy, proposed improvements, and willingness to sustain and scale it (Multimedia Appendix 4).

#### Health Care Pathway and Support From CBOs

At every stage of study, The National Centre for HIV/AIDS Dermatology and STDs and CBOs who are closely working with MSM and TGWs will help define selected criteria, identify the seeds, localize their hot spots, and promote the study. The National Centre for HIV/AIDS Dermatology and STDs will also help review the tutorials for the HIV self-test device and the promotional messages and will organize consultative meetings with stakeholders. The CBOs, through community health workers, will coordinate the work between pharmacists and participants and counsel the participants regarding the necessary follow-up after returning the HIV self-test results.

According to the results, they will provide the participants with proposals for support for confirmatory tests, to ART clinics in case of a positive reaction, or to PrEP services in the case of a negative reaction.

#### Sample Collection and Tests

The HIV self-test used in the study is OraQuick, which is a private and rapid HIV test using oral fluid with safe, accurate, and quick results.

The OraQuick HIV self-test uses oral fluid to check for HIV-1 and HIV-2 antibodies. It provides results in about 20 minutes and can detect the virus in more than 99% of people who are infected with HIV. Because the test is a “screening” test, a second test to confirm the results is always advised, and participants will be referred for that purpose in case of a reactive HIV self-test.

#### Loss to Follow-Up

When a participant who has not explicitly withdrawn consent does not return the HIV self-test result after 1 week, the research team will reach them by phone. In addition, 1 week after returning positive HIV self-test results, if there is no information confirming the linkage to one of the partner ART sites or participants do not show up after receiving the reminder for a 6-month HIV self-test, the research team will reach them by phone.

A participant who does not show up for a given scheduled visit and for whom the study team fails to reach by phone will be considered lost to follow-up.

### Analysis

#### Primary Outcome

The percentage (95% CI) of HIV self-tests delivered among the total number of coupons distributed will be determined and stratified by gender (MSM or TGW) and type of coupon (paper or electronic version).

#### Secondary Outcomes

In addition, the data collected from the study will enable estimation of the percentage (95% CI) of HIV self-test results recorded among the total number of HIV self-tests delivered and stratified by gender (MSM or TGW); for those contacted by phone, the percentage (95% CI) of HIV self-tests realized among the number of nonrecorded HIV test results; the percentage (95% CI) of participants with positive HIV self-tests linked to an ART site and stratified by gender (MSM or TGW); the percentage (95% CI) of participants with negative HIV self-tests linked to PrEP services and stratified by gender (MSM or TGW); the percentage (95% CI) of participants with 6-monthly repeated HIV testing during the 18 months; and the probability (95% CI) of HIV testing discontinuation as well as the median time to the occurrence of HIV testing discontinuation. The latter will be determined using Kaplan Meier analysis, to take into account censored data due to loss of follow-up.

Data from the questionnaires and HIV results will be obtained from a data server in a Microsoft Excel file and imported and appended into a single matrix using Stata version 18SE (StataCorp). We will describe the characteristics of key populations with summary statistics (proportions, 95% CIs, measures of central tendency), which will be analyzed using descriptive statistics with a significance level of .05.

The proportions of the key populations who receive the HIV self-test kit from the pharmacy and those who complete the test are key elements for informing the scaling of the intervention.

#### Qualitative Study

For the qualitative assessment, bilingual research staff will transcribe all interviews verbatim and translate them into English. All transcripts will be analyzed according to the 6-phase reflexive thematic approach by Braun and Clarke [39]. Two independent coders will thoroughly review the transcripts and apply open coding to generate initial codes. Subthemes will be directly identified based on transcripts rather than relying on pre-existing concepts. In axial coding, the codes will then be compared and organized into potential themes, which will then be assessed against the extracted codes and further refined until no more emergent themes are found. Any discrepancies in coding will be resolved through iterative discussions with a third research member. QSR NVivo V.14 for Windows will be used for data management.

## Results

In this research project, we anticipate that the implementation of HIV self-testing through private pharmacy networks combined with a respondent-driven sampling strategy will significantly enhance HIV testing uptake among MSM and TGW in Phnom Penh, Cambodia. By providing a confidential, anonymous, and convenient testing option, we expect an increase in the number of individuals who know their HIV status, thereby facilitating early diagnosis and linkage to care. Additionally, we aim to estimate the uptake of HIV self-testing among MSM and TGW. Thanks to respondent-driven sampling and selection criteria that diversify the seeds, the study may lead us to identify new profiles or characteristics of MSM and TGW at risk of HIV acquisition compared with those described in the latest IBBS report. Furthermore, self-stigma among MSM and TGW and their willingness to repeat the HIV self-test every 6 months will be evaluated. Through focus group discussions, we will obtain insights into the perception of the appropriateness, acceptability, barriers, and facilitators of free HIV self-test distribution at private pharmacies via the respondent-driven sampling technique from both pharmacists (providers) and MSM and TGW (users). The innovative use of respondent-driven sampling is predicted to reach previously unidentified networks within key populations, potentially uncovering new cases and further broadening the impact of the intervention. Collectively, these results will constitute key elements to inform the scaling of the intervention policy in the country. Last but not least, the expected outcomes of this project are in line with the broader goals of reducing HIV transmission rates and improving health outcomes for MSM and TGW in southeast Asia, contributing to the global effort to curb the HIV/AIDS epidemic.

To date, significant progress has been made in preparation for the study’s implementation. The study was funded by Agence nationale de recherches sur le sida, les hépatites, et les maladies infectieuses émergentes (ANRS; grant number: ECTZ176682) in September 2023. Approval of the study protocol was successfully obtained from the NECHR in Cambodia and the Commission Nationale de l'Informatique et des Libertés (CNIL) in France (Autorisation Tacite) in February 2025. An operation manual and respondent-driven sampling monitoring system have been designed to facilitate the implementation of the respondent-driven sampling methodology, and comprehensive preparations are underway to ensure project launch. The schedule of key activities is shown in Table 1. Data collection will be conducted between September 2024 and December 2025. The initial results are expected to be published in February 2026.

**Table 1:** Timeline of key activities of the study and expected completion duration.

Activities	Estimated duration
Project launch	Month 1
Tool development	Months 1-5
Consultative meeting	Month 1
Data collection and follow-up	Months 4-21
Qualitative study	Months 9-11
Transcription and translation	Months 9-12
Data analysis and reporting	Months 13-15 (qualitative analysis) and months 16-23 (quantitative analysis)
Dissemination	Month 24

## Discussion

### Overview

Routine HIV testing is one of the cornerstones of efficient HIV management. This is particularly relevant in light of the persistently high HIV incidence among TGW and MSM in Asian countries, where stigma and discrimination can be significant barriers to accessing HIV services. Despite the expansion of testing coverage and the introduction of innovative strategies like PrEP, the ongoing stigmatization of sexual activity among men and the concealed nature of such interactions exacerbate the challenge of curbing new infections [6]. To reduce new infections (11,000 people living with HIV/AIDS were unaware of their status in Cambodia in 2022), screening programs must achieve the broadest possible coverage. Screening coverage remains insufficient for MSM and TGW. For example, in the Global Fund Funding Cycle 2024-2026, the target of 93,985 MSM is fair for the program’s reach of 50,000 [40]. Furthermore, HIV/AIDS awareness among those aged 15 years to 24 years is critically low, at only 23% to 27%. This highlights the urgent need for expanded education and screening about HIV among young people. Therefore, maximizing testing coverage to achieve the first 95 targets as quickly as possible is the top priority. This situation underscores the necessity for new privacy-centric and stigma-reducing approaches.

This study’s investigation into the deployment of HIV self-testing via private pharmacies combined with the respondent-driven sampling strategy presents a viable path to enhance testing uptake within hard-to-reach cohorts. Using private pharmacies aims to offer a discreet and accessible option for HIV testing, potentially navigating around the stigma and discrimination barriers. Furthermore, using respondent-driven sampling aims to leverage the social networks of MSM and TGW, reaching individuals less likely to use conventional health services, including those who do not self-identify as gay, thereby extending the reach to bridge populations [41,42].

This approach’s success hinges on various factors, including pharmacy participation, HIV self-testing’s acceptability within target groups, and respondent-driven sampling’s capacity to yield representative samples. Should this model prove efficacious, its scalability could facilitate broader implementation within Cambodia and regions with similar epidemiological contexts.

In the MSM and TGW communities, community-based testing is promoted and well-accepted. Indeed, screening conducted by CBOs has proven to be the most effective method for identifying new infections within these communities [5,43]. A critical aspect of this project is assessing the acceptability of pharmacy-based testing among key populations and to understand how these communities embrace and potentially promote this testing approach.

Moreover, the study seeks to shed light on contemporary risk factors like chemsex, which is becoming increasingly relevant in discussions of HIV transmission among MSM [5]. By exploring these new dimensions, the research could contribute valuable insights into the complex interplay of social behaviors and HIV risk, further enhancing our understanding of the dynamics of the epidemic.

It will also address the potential challenges of late diagnosis and testing service underutilization by key demographics, highlighted by the disparities in HIV awareness and testing uptake across the country. The strategy aims to bridge these gaps, providing a pathway to bolster the initial stage of the HIV care continuum.

It is important to acknowledge the study’s limitations, including the potential biases inherent in self-reported data and concerns regarding the findings’ generalizability. Nevertheless, the outcomes of this research hold the promise of informing future public health initiatives, proposing a framework for incorporating HIV self-testing into a broader HIV prevention and care strategy focused on privacy, accessibility, and community engagement. This could notably contribute to the identification and engagement of bridge populations and the study of emerging risk factors within the context of HIV transmission.

### Limitations

Our study faces specific limitations as well. The use of a respondent-driven sampling method based on paper and electronic vouchers, although innovative, might not capture the full diversity of the MSM and TGW populations, possibly resulting in skewed data. The stigma surrounding HIV and marginalized groups may also impact participation rates and individuals’ willingness to disclose sensitive information. Excluding data from individuals who decline to participate in this study introduces a potential selection bias particularly in terms of understanding barriers to free HIV self-test distribution at private pharmacies. Moreover, distributing HIV self-test kits through private pharmacies, despite the convenience and guaranteed privacy and anonymity, might not reach individuals who are geographically, economically, or socially distanced from these facilities. Furthermore, recruitment will focus on hot spots and social networks in Phnom Penh, excluding, unfortunately, for legal reasons, younger MSM and TGW younger than 18 years. Preferences for these platforms may shift over time. Consultative meetings with CBOs and stakeholders, who are working closely with these communities, will aid in refining our outreach strategies.

These challenges call for a careful interpretation of our findings and highlight the need for additional investigation to achieve a more comprehensive understanding of HIV testing behaviors within these key populations.

### Conclusion

This research project aims to address the high HIV rates among MSM and TGW in southeast Asia by introducing HIV self-testing through private pharmacies and using respondent-driven sampling to reach these marginalized groups. The feasibility of this approach would increase HIV testing uptake, detect new infections, and enhance the initial step of the HIV care cascade. Positive findings could serve as a basis for considering the expansion of this model to more pharmacies across the country, setting a precedent for future interventions and research in similar contexts.

The project also emphasizes a collaborative effort with community organizations and community workers, representing a public-private partnership to ensure a respectful, high-quality, and customized testing experience, thereby overcoming traditional barriers to HIV testing such as stigma and discrimination. The insights gained from this study are expected to contribute to the broader public health strategy against HIV/AIDS, offering evidence-based recommendations for policy and practice not only in Cambodia but potentially in other regions with analogous epidemiological and social landscapes.

## Appendix

Multimedia Appendix 1 Questionnaire for pilot implementation of Hiv self-testing delivery in private pharmacies combined to a respondent driven sampling method to improve Hiv testing for men who have sex with men (MSM) and transgender women (TGW) in Phnom Penh.

Multimedia Appendix 2 Topic guide for focus group among men who have sex with men and transgender women.

Multimedia Appendix 3 Topic guide for group discussion with pharmacists.

Multimedia Appendix 4 Interview guide for key informants.

## Abbreviations

ANRS: Agence nationale de recherches sur le sida, les hépatites, et les maladies infectieuses émergentes
ART: antiretroviral therapy
CBO: community-based organization
CNIL: Commission Nationale de l'Informatique et des Libertés
IBBS: Integrated Biological and Behavioral Survey
MSM: men who have sex with men
NECHR: National Ethics Committee for Health Research
PrEP: preexposure prophylaxis
TGW: transgender women
WHO: World Health Organization

## Data Availability

Data sharing is not applicable to this manuscript since no data sets were generated nor analyzed during the development of the study protocol described herein. Once the data collection begins, all investigators will have access to anonymized patient-level data.
