# Supplementary material for: Pilot Implementation of HIV Self-Testing Delivery in Private Pharmacies Combined With a Respondent-Driven Sampling Method to Improve HIV Testing for Men Who Have Sex With Men and Transgender Women in Phnom Penh (ANRS 0100s): Protocol for a Prospective Mixed Method Feasibility Study
Source: JMIR Res Protoc. 2025 Jun 27;14:e65351. doi: 10.2196/65351 (PMC12254708; doi:10.2196/65351)
Supplement: Multimedia Appendix 1 [file resprot_v14i1e65351_app1.pdf]

Electronic Case Report Form

''Pilot implementation of HIV self-testing delivery in private pharmacies combined to a Respondent Driven Sampling method to improve HIV testing for MSM and TGW in Phnom Penh –ANRS 0100s''

Version 1.2, 05th April 2024

General instruction

- All the response methods are explained explicitly to the respondent
- When the answer is ''Others, please specify'', fill with appropriate text inputs!
- If the respondent needs advice or support, refer to community worker at place

Consent and eligibility

1. How do you self-identify your gender as?
   - ☐ Transgender woman (TGW) ''you were born as a male but define yourself as a woman or third gender'' → move to question 3
   - ☐ Cisgender man ''you were born as a male and define yourself as a man) → the participant is excluded from the study
   - ☐ Transgender man ''you were born as a female and define yourself as a man'' → the participant is excluded from the study
   - ☐ Cisgender women ''you were born as a female and define yourself as a woman'' → the participant is excluded from the study
2. Have you had at least one oral or anal intercourse with another man in the past 12 months?
   - ☐ Yes
   - ☐ No → the participant is excluded from the study
3. Have you had at least one oral, anal or vaginal intercourse with another man in the past 12 months?
   - ☐ Yes
   - ☐ No → the participant is excluded from the study
4. What is your known HIV status?
   - ☐ Positive → the participant is excluded from the study
   - ☐ Negative
   - ☐ Not sure

5. How old are you?

|__|__| years old → if younger than 18 years old by the question date, the participant is excluded from the study.

6. Have you provided the confirmation of participation ‘’Yes’’ in the e-consent form?
    - ☐ Yes
    - ☐ No → the participant is excluded from the study
7. Can you read khmer?
    - ☐ Yes (skip question 8)
    - ☐ No
8. In case you cannot read khmer, do you consent that CBW or clinical research assitant help fill in the quesitionnaire for you?
    - ☐ Yes
    - ☐ No
9. During last month, in the region you are living how many people are there who have similar sexual orientation?
    - ☐ Yes
    - ☐ No

Study location and participant's information

Partcipant's code: |__|__|__|__|

Participation date (dd/mm/yyyy): |__|__|/|__|__|/|__| |__|__| __|

Pharmacy location in Phnom Penh where you received the test:

- ☐ Outlet 1 address: .... Phone: ....
- ☐ Outlet 2 address: .... Phone: ....
- ☐ Outlet 3 address: .... Phone: ....
- ☐ Outlet 4 address: .... Phone: ....
- ☐ Outlet 5 address: .... Phone: ....

Part I: Sociodemographic information, gender identity

---

10. Do you speak khmer?
    - ☐ Yes
    - ☐ No
11. Are you currently in a stable relationship whatever you are living with this person or not?
    - ☐ Yes with man
    - ☐ Yes with woman

- ☐ No
- ☐ Refused to answer

12. What was your last years of formal education have you completed?
    - ☐ Never attended the school
    - ☐ Primary
    - ☐ High school
    - ☐ University
13. What is your main occupation?
    - ☐ Hair dresser/beautician
    - ☐ Officer worker
    - ☐ Labourer/farmer
    - ☐ Seller
    - ☐ Student
    - ☐ Other, please specify______________________
14. What is your monthly income (in USD)? (if you are not sure, just provide us the approximative amount)
    - ☐ No income
    - ☐ <100 USD per month
    - ☐ 100 – 199 USD per month
    - ☐ 200 – 299 USD per month
    - ☐ ≥300 USD per month
15. What was your sex at birth?
    - ☐ Male
    - ☐ Female
16. How would you define your gender today?
    - ☐ Man
    - ☐ Woman
    - ☐ Non-binary
17. How would you self-define your sexual orientation?
    - ☐ Gay or homosexual
    - ☐ Bisexual
    - ☐ Straight or heterosexual
    - ☐ Pansexual
    - ☐ Others please specify________

Part II: Sexual behavior

---

18. Do you consider yourself as being?:
    - ☐ Very exposed to HIV
    - ☐ Rather exposed to HIV

- ☐ Few exposed to VIH
- ☐ Not at all exposed to HIV

19. How many different sexual partners did you have in the last 6 months? (Please consider all persons you had sex with, regardless of the type of relationship you had with them. If you do not know exactly, please provide an estimate.)

|__|__| male sexual partners

|__|__| female sexual partners

20. Over the last 6 months, how often did you have insertive anal sex (being top) with a man?
    - ☐ Never
    - ☐ Sometimes
    - ☐ Often
    - ☐ Always
21. Regarding insertive anal sex, did you use condoms?
    - ☐ Systematically
    - ☐ Most of the times
    - ☐ Sometimes
    - ☐ Never
22. Over the last 6 months, how often did you have receptive anal sex (being bottom) with a man?
    - ☐ Never
    - ☐ Sometimes
    - ☐ Often
    - ☐ Always
23. Regarding receptive anal sex, did you use condoms?
    - ☐ Systematically
    - ☐ Most of the time
    - ☐ Sometimes
    - ☐ Never
24. Over the last 6 months, how often did you have vaginal sex?
    - ☐ Never
    - ☐ Sometimes
    - ☐ Often
    - ☐ Always
25. Regarding vaginal sex, did you use condoms?
    - ☐ Systematically
    - ☐ Most of the times
    - ☐ Sometimes
    - ☐ Never

26. How did you meet your male sexual partners in the last 6 months? (multiple answers are allowed)
    - Street-based hotspots
    - Entertainment-based hotspots including bars, clubs
    - Sauna, massage
    - Colleagues or friends
    - Internet website
    - Social networks (facebook, twitter…)
    - Dating applications
27. Which dating applications did you use in the last 6 months? (multiple answers are allowed)
    - I don't use any application
    - Blued
    - Grinder
    - Hornet
    - Jack'd
    - Tinder
    - Scruff
    - Wechat
    - Others please specify__________
28. In the last 6 months, did you receive money, goods or services in exchange of sex?
    - Yes
    - No → move to question 30
29. If yes, how many clients?

|__|__| male clients

|__|__| female clients including transgender women

30. In the last 6 months, did you spend money, goods or services in exchange of sex?
    - Yes
    - No → move to question 32
31. If yes, how many sex providers?

|__|__| male sex providers

|__|__| female sex providers including transgender women

Part III: HIV testing

---

32. When did you last test for HIV?
    - Never tested → if never, move to question 35
    - Last six months
    - 7-12 months
    - 13-36 months

- ☐ 37 months or more (more than 3 years ago)

33. The last time you had an HIV test, where was it done?
    - ☐ Private facility
    - ☐ Public facility
    - ☐ NGO facility
    - ☐ NGO outreach worker
    - ☐ Others please specify__________
34. How was your last time HIV test realised?
    - ☐ Venipuncture blood sample performed by CBO staffs or nurses
    - ☐ Blood finger-prick performed by CBO staffs or nurses
    - ☐ Blood finger-prick performed by yourself
    - ☐ Salivary test performed by CBO staffs or nurses
    - ☐ Salivary test performed by yourself
35. If HIV self-test is provided free in form of finger-prick blood test, how much are you willing to accept this?
    - ☐ Strongly unaccept
    - ☐ Unaccept
    - ☐ Uncertain/neutral
    - ☐ Accept
    - ☐ Strongly accept
36. If HIV self-test is provided along with syphilis test in form of finger-prick blood, how much are you willing to accept this?
    - ☐ Strongly unaccept
    - ☐ Unaccept
    - ☐ Uncertain/neutral
    - ☐ Accept
    - ☐ Strongly accept

Part IV: Substance use (alcohol and drug use)

---

37. Have you had a Binge drink (drank six or more drinks on one occasion) in the past 6 months?

- ☐ Never
- ☐ Less than once a month
- ☐ Once a month
- ☐ Once a week
- ☐ Almost or every day

38. Have you felt you ought to cut down your consumption of alcholic drinks ?

- ☐ Yes
- ☐ No

39. Has anyone around you ever commented on your drinking?

☐ Yes
☐ No

40. Have you ever felt that you were drinking too much?
    ☐ Yes
    ☐ No
41. Have you ever needed alcohol first in the morning?
    ☐ Yes
    ☐ No

42. Have you used any drug in the past 6 months?

☐ No → move to question 44
☐ Yes

43. If yes, what drugs did you use? (multiple answers are allowed you)

**Drug Name**	**Use**	**Injection**	**Used for Chemsex**
Metamphetamin	☐	☐	☐
Mephedrone (meow/meph)	☐	☐	☐
GHB/GBL	☐	☐	☐
Ketamine	☐	☐	☐
Cannabis	☐	☐	☐
Other please specify ____________	☐	☐	☐

Part VI: Self-stigma using self-stigma scale–short form (SSS–S)

44. For each item, please tick for answer with scale that matches you the most

**Items**	**Strongly disagree**	**Disagree**	**Agree**	**Strongly agree**
Cognitive				
My identity as a MSM/TGW is a burden to me				
My identity as a MSM/TGW incurs inconvenience in my daily life				
The identity of being a MSM/TGW taints my life				
Affective				
I feel uncomfortable because I am a MSM/TGW				

I fear that others would know that I am a MSM/TGW				
I feel like I cannot do anything about my MSM/TGW status				
Behavioral				
I estrange myself from others because I am a MSM/TGW				
I avoid interacting with others because I am a MSM/TGW				
I dare not to make new friends lest they find out that I am a MSM/TGW				

Part VI: Access to HIV prevention program

---

45. Did you any receive items/services from HIV prevention programme in the last 6 month? (multiple answers are allowed)

☐ Nothing
☐ Condoms
☐ Lubricant
☐ Pamphlet or brochure
☐ STI services
☐ PrEP services
☐ Other medical services
☐ HIV testing
☐ Training on condom use
☐ Counselling on risk
☐ Referral

46. Are you currently on PrEP?

☐ Yes, on demand PrEP
☐ Yes, daily PrEP
☐ No. I never used PrEP
☐ No. I stop using PrEP
☐ No. I don't know what PrEP is

47. Was the questionnaire administered by?

☐ CBO
☐ Self-administered

THE END
