# Supplementary material for: Pilot Implementation of HIV Self-Testing Delivery in Private Pharmacies Combined With a Respondent-Driven Sampling Method to Improve HIV Testing for Men Who Have Sex With Men and Transgender Women in Phnom Penh (ANRS 0100s): Protocol for a Prospective Mixed Method Feasibility Study
Source: JMIR Res Protoc. 2025 Jun 27;14:e65351. doi: 10.2196/65351 (PMC12254708; doi:10.2196/65351)
Supplement: Multimedia Appendix 2 [file resprot_v14i1e65351_app2.pdf]

Guide for Focus Group Discussion ''MSM and TGW''

'' Pilot implementation of HIV self-testing delivery in private pharmacies combined to a Respondent Driven Sampling method to improve HIV testing for MSM and TGW in Phnom Penh – ANRS 0100s''

- Introduction
- Greet participants
- Thanks for their time
- Explain about the objectives of the study
- Explain about the information sheet and take consent
- Ask 1 to 2 icebreaking questions
    - Have you ever tested for HIV? (if no, why?)

**Discussion Topics**	**Question Guide**
**Perception, Acceptability and appropriateness**	1. In your view, what are the advantages of this oral HIV self-testing (HIVST)?<br>Probe for:<br>• User's experience on HIVST<br>• Advantages<br><br>2. In your view, what are the disadvantages of this oral HIV self-testing (HIVST)?<br>Probe for:<br>• Disadvantages (concern…)<br><br>3. Do you have any difficulties in realising HIVST? Are the instructions for use easy to understand?<br>Probe for:<br>• List of difficulties encountered during the test realization<br><br>4. Do you have any anxieties about performing HIVST test alone and interpreting HIVST results? Why?<br>Probe for:<br>• Concern or anxieties of realising and interpreting the results alone<br><br>5. What are your thoughts on private pharmacy supplying the HIVST for free? Why do you say so? (Do you think the private pharmacy can be a relevant distribution point for delivering free HIVST? Why?)<br>Probe for:<br>• Perception on free delivering HIVST by private pharmacy<br>• Advantages<br>• Disadvantages<br><br>6. If the private pharmacy really dispenses the HIVST, what would be your suggestion for optimal delivery?

	7. Up to which price (USD) will you be able to buy HIVST on your own?<br>Probe for:<br>• maximum price they could afford
**HIVST use and experience: facilitators and barriers**	8. Have you been hesitant to conduct HIVST? Why?<br>Probe for:<br>• Barriers<br><br>9. What factors drove you to conduct HIVST?<br>Probe for:<br>• Facilitators
**Promoting HIVST**	10. Is it difficult to promote the HIVST to others? Why? Are there any differences according the types of friends/partners?<br><br>11. From your personal experience, to whom you gave the coupons?
**Willingness to repeat the HIVST**	12. Are you willing to conduct HIVST every 6 months? Why?

____
