# Supplementary material for: Pilot Implementation of HIV Self-Testing Delivery in Private Pharmacies Combined With a Respondent-Driven Sampling Method to Improve HIV Testing for Men Who Have Sex With Men and Transgender Women in Phnom Penh (ANRS 0100s): Protocol for a Prospective Mixed Method Feasibility Study
Source: JMIR Res Protoc. 2025 Jun 27;14:e65351. doi: 10.2196/65351 (PMC12254708; doi:10.2196/65351)
Supplement: Multimedia Appendix 3 [file resprot_v14i1e65351_app3.pdf]

<u>**Guide for Focus Group Discussion ''Pharmacists''**</u>

'' Pilot implementation of HIV self-testing delivery in private pharmacies combined to a Respondent Driven Sampling method to improve HIV testing for MSM and TGW in Phnom Penh – ANRS 0100s''

- Introduction
- Greet participants
- Thanks for their time
- Explain about the objectives of the study
- Explain about the information sheet and take consent
- Ask 1 or 2 icebreaking questions

Discussion Topics	Questions Guide
**Perception, acceptability and appropriateness**	1. In your point of view, what are the advantages<br>Probe for:<br>• Advantages of delivering HIVST in general by the pharmacy<br><br>2. In your point of view, what are the disadvantages of delivering self-HIV testing (HIVST) with private pharmacy?<br>Probe for:<br>• Disadvantages (concern…)<br>3. How is it perceived by your clients?<br>Probe for:<br>• Feedbacks from clients<br><br>4. What are your thoughts on private pharmacy supplying the HIVST for free to MSM and TGW? Why do you say so?<br>Probe for:<br>• Perception on free delivering HIVST by private pharmacy to specific populations: MSM and TGW<br>• Advantages<br>• Disadvantages including logistic issues<br><br>5. How much does distributing HIVST to MSM/TGW increase your current workload? Are you able to handle this or is it a real burden?<br>• Workload/extra-burden<br><br>6. What are your thoughts on peer-recruitment (RDS) process used in the study? Why do you say so?<br>Probe for:<br>• Perception on the RDS (electronic coupon, paper coupon…)

**Willingness to sustain the strategy and improvement**	1. For you, what would be the optimal price of HIVST (for clients, and for pharmacists)? Why?<br>Probe for:<br>• Price with acceptable incomes from the perspective of clients<br>• Price with the minimum benefits acceptable from the point of view by pharmacists<br><br>2. If the private pharmacy really dispenses the HIVST, what would be your suggestion for optimal delivery?<br><br>3. Do you think other than private pharmacy are there any channels to distribute HIVST or increase the HIVST testing among MSM and TGW?<br><br>Probe for:<br>• Other channel beside ''private pharmacy using RDS to reach MSM and TGW''.<br><br>4. What would be your suggestion to the recruitment process in order to reach more MSM-TGW networks to come to get the HIVST from the pharmacy?
