# Supplementary material for: Pilot Implementation of HIV Self-Testing Delivery in Private Pharmacies Combined With a Respondent-Driven Sampling Method to Improve HIV Testing for Men Who Have Sex With Men and Transgender Women in Phnom Penh (ANRS 0100s): Protocol for a Prospective Mixed Method Feasibility Study
Source: JMIR Res Protoc. 2025 Jun 27;14:e65351. doi: 10.2196/65351 (PMC12254708; doi:10.2196/65351)
Supplement: Multimedia Appendix 4 [file resprot_v14i1e65351_app4.pdf]

Guide for Key Informant Interview for Policymakers

'' Pilot implementation of HIV self-testing delivery in private pharmacies combined to a Respondent Driven Sampling method to improve HIV testing for MSM and TGW in Phnom Penh – ANRS 0100s''

Version 1.2, 05th April 2024

- Introduction
- Greet participant
- Thank for the time
- Explain about the objectives of the study
- Explain about the information sheet and take consent
- Ask 1 or 2 icebreaking questions

Discussion Topics	Questions Guide
**Perception, acceptability and appropriateness**	1. In your point of view, what are the advantages of delivering self-HIV testing (HIVST) with private pharmacy?<br>Probe for:<br>• Advantages of delivering HIVST in general by the pharmacy<br><br>2. In your point of view, what are the disadvantages of delivering self-HIV testing (HIVST) with private pharmacy?<br>Probe for:<br>• Disadvantages (concern…)<br>3. What are your thoughts on private pharmacy supplying the HIVST for free to MSM and TGW? Why do you say so?<br>Probe for:<br>• Perception on free delivering HIVST by private pharmacy to specific populations: MSM and TGW<br>• Advantages<br>• Disadvantages including logistic issues<br><br>4. What are your thoughts on peer-recruitment (RDS) process used in the study? Why do you say so?<br>Probe for:<br>• Perception on the RDS (electronic coupon, paper coupon…)<br>5. Do you have any other suggestion or remarks to this strategy (free distribution of HIVST from private pharmacy to MSM & TGW)?

**Willingness to sustain the strategy and improvement**	1. In your own opinion, how to sustain the HIV self-test kits from private pharmacies for free to MSM & TGW?<br>Probe for:<br>• Favorable factors/opportunities<br>2. If free distribution is challenging, for you, what would be the optimal price of HIVST (for clients, and for pharmacists)? Why?<br>Probe for:<br>• Price with acceptable incomes from the perspective of clients<br>• Price with the minimum benefits acceptable from the point of view by pharmacists<br>3. If the private pharmacy really dispenses the HIVST for free, what would be your suggestion for optimal delivery?<br>4. What would be your suggestion to the RDS recruitment process in order to reach more MSM-TGW networks to come to get the HIVST from the pharmacy?<br>5. Do you think other than private pharmacy are there any channels to distribute HIVST among MSM and TGW?<br>Probe for:<br>• Other channel beside ''private pharmacy using RDS to reach MSM and TGW''.<br>6. Do you have other remark or concern you would like to share?

___
